# Plant-Based Alternatives to Mold-Ripened Cheeses as an Innovation among Dairy Analogues

**DOI:** 10.3390/foods13142305

**Published:** 2024-07-22

**Authors:** Agata Fabiszewska, Katarzyna Wierzchowska, Ilona Dębkowska, Weronika Śliczniak, Magdalena Ziółkowska, Karina Jasińska, Joanna Kobus, Dorota Nowak, Bartłomiej Zieniuk

**Affiliations:** 1 Department of Chemistry, Institute of Food Sciences, Warsaw University of Life Sciences-SGGW, 159c Nowoursynowska St., 02-776 Warsaw, Poland; agata_fabiszewska@sggw.edu.pl (A.F.); karina_jasinska@sggw.edu.pl (K.J.); bartlomiej_zieniuk@sggw.edu.pl (B.Z.); 2 Faculty of Biology and Biotechnology, Warsaw University of Life Sciences-SGGW, 159 Nowoursynowska St., 02-776 Warsaw, Poland; ilona.debkowska@sggw.edu.pl (I.D.); s200167@sggw.edu.pl (W.Ś.); s200179@sggw.edu.pl (M.Z.); 3 Faculty of Food Technology, Warsaw University of Life Sciences-SGGW, 159c Nowoursynowska St., 02-776 Warsaw, Poland; s201911@sggw.edu.pl; 4 Department of Food Engineering and Process Management, Institute of Food Sciences, Warsaw University of Life Sciences-SGGW, 159c Nowoursynowska St., 02-776 Warsaw, Poland; dorota_nowak@sggw.edu.pl

**Keywords:** vegan cheese analogue, plant-based alternative, cashews, mold, LAB, novel food

## Abstract

There is a growing demand for vegan products and plant-based food when dealing with the impact of livestock on the climate crisis. The aim of this study was to develop a formulation for a plant-based analogue of mold-ripened cheese. Were investigated the following plant materials: cashews, pistachios, soy flour, chickpea flour, pea protein, pumpkin protein, hemp protein, and spirulina powder. Plant matrices were fermented with lactic acid bacteria (LAB) starter cultures and cheese starter cultures of mold species *Geotrichum candidum* and *Penicillium camemberti*. All microorganisms’ growth were tested in a vegan-type culture medium. Calcium supplementation was applied and followed by an in-depth analysis of the elemental composition of selected analogues with inductively coupled plasma optical emission spectroscopy. The physicochemical and organoleptic analyses of plant-based alternatives of Camembert were conducted. This is the first paper describing novel formulations for plant-based alternatives for Camembert cheese prepared with techniques mimicking the original milk product.

## 1. Introduction

In recent years, the market of vegan products, including vegan cheeses, has undergone a significant transformation. A mere few years ago, access to these products was severely limited, with vegan cheeses failing to emulate the taste and consistency of their dairy counterparts. Presently, these plant-based products have achieved the desired characteristics: a velvety texture, easy melting, and a more natural flavor. Concurrently, there is a growing interest in plant-based cheeses crafted using traditional techniques, driven by a multitude of factors, including environmental concerns, ethical considerations, and dietary preferences [1].

A cheese analogue is a complex product resembling cheese that is crafted by fully or partially substituting animal-based ingredients such as milk, milk fat, or milk protein with plant-based alternatives. The origins of plant-based cheese alternatives can be traced back to ancient civilizations, where fermented nut and seed-based products were consumed as a means of preserving and enhancing flavors [2]. However, it was not until the late 20th century that the modern plant-based cheese industry began to take shape, driven by the growing demand for dairy-free alternatives and advancements in food technology [3].

Among various dairy analogues, plant-based alternatives to mold-ripened cheeses have emerged as a particularly intriguing and challenging endeavor. These innovative products aim to replicate the distinctive flavors, textures, and mold-ripened characteristics of their dairy counterparts while adhering to vegan and sustainable development [4]. Contemporary developments enable the production of cheese analogues with enhanced nutritional value at reduced costs. Consumers are increasingly gravitating towards “low-fat” or “cholesterol-free” products, prompting the industry to cater to these evolving preferences [5]. These substitutes address concerns about hormones and antibiotic residues, are also “free from” cholesterol, lactose, and dairy allergens, and feature vegan-friendly labels [6]. They are characterized by high vitamin and mineral content, along with added functionalities like dietary fiber and pre- or probiotic effects. The growing popularity has led to a diverse range of plant matrices in these products [7].

The foundation of plant-based cheese analogues lies in the selection of suitable plant matrices, which serve as the primary source of proteins, fats, and other functional ingredients. These matrices are carefully chosen and combined to achieve the desired texture, flavor, and melting properties of the final product. Almonds, cashews, macadamia nuts, and seeds such as sunflower and pumpkin seeds are commonly used as protein sources in plant-based cheese analogues [8]. Moreover, soybeans, peas, and other lentils are rich in proteins and have been extensively utilized in the formulation of plant-based alternatives [9]. Various vegetable oils (coconut, palm, or sunflower), starches, and polysaccharides (carrageenan, xanthan gum, and guar gum) are employed to enhance the texture, stability, and melting properties of plant-based alternative food products [8,9]. Above all, replicating the unique characteristics of mold-ripened cheeses presents a significant challenge for manufacturers because traditional mold-ripened cheeses, such as Brie, Camembert, and Roquefort, derive their distinctive flavors and textures from the action of specific mold species, primarily *Penicillium camemberti* and *P. roqueforti* and lactic acid bacteria (LAB).

Therefore, the current paper focused on formulating a formulation for developing a plant-based alternative to ripening cheese utilizing molds and LAB. The specific objectives also included comparing the growth of strains of LAB and filamentous fungi in culture media that do not contain animal ingredients. Additionally, the objectives involved assessing the possibility of using selected plant matrices to produce an analogue of long-ripened cheese and developing a method for fortifying plant cheese analogues with calcium.

## 2. Materials and Methods

### 2.1. Materials

This project utilized the following materials: cashews and pistachio nuts purchased from the local supermarket in Warsaw (Poland), as well as soy and chickpea flours (Targroch, Filipowice, Poland), pea, pumpkin, hemp protein powders, and powdered spirulina (Bio Planet S.A., Leszno, Poland). The Vzyme preparation of liquid transglutaminase for texturizing applications was purchased from Cashewbert (Berlin, Germany). All chemicals used within this study were purchased from Avantor Performance Materials Poland S.A. (Gliwice, Poland) and Sigma-Aldrich (Poznan, Poland).

### 2.2. Biological Material

In the experiments, the following strains were used: *Streptococcus salivarius* KKP 3251 and *Lactococcus lactis* KKP 3020 from the Collection of Industrial Microorganisms of the Prof. Waclaw Dabrowski Institute of Agricultural and Food Biotechnology—State Research Institute (Warsaw, Poland), as well as cultures purchased from Cashewbert (Berlin, Germany), i.e., “Mesophilic Starter Cultures” comprising *S. thermophilus*, *L. lactis* subsp. *cremoris*, and *L. lactis* subsp. *lactis*, “Thermophilic Cheese Starter for fresh and aged cheese” with *S. thermophilus* and *L. helveticus*, “*Geotrichum candidum* powder”, and “Traditional strain of *P. candidum*” (*P. camemberti*).

### 2.3. Culture Media

Bacteria were cultured on two types of liquid media, namely MRS (Graso Biotech, Owidz, Poland) and MRS Broth modified Vegitone (Sigma-Aldrich, Poznań, Poland), containing plant peptone instead of animal peptone, with pH = 6.6, and solid media MRS agar, M17 agar, and MRS Vegitone Agar solidified with agar at a concentration of 20 g/dm^3^. In the case of liquid media for mold growth, Vegitone infusion broth (Sigma-Aldrich) with a pH of 5 was used, and the reference medium consisted of the following: NH_4_NO_3_—1 g/dm^3^, (NH_4_)_2_SO_4_—1 g/dm^3^, K_2_HPO_4_—4 g/dm^3^, KH_2_PO_4_—2 g/dm^3^, NaCl—1 g/dm^3^, yeast extract—1 g/dm^3^, and glucose—10 g/dm^3^, with pH = 5 adjusted with a 10% solution of citric acid. Solid media for fungi included Vegitone infusion with the addition of agar (20 g/dm^3^) and DRBC (Dichloran-Rose Bengal Chloramphenicol agar, Graso Biotech).

### 2.4. Microorganism Culture Conditions and Evaluation of Medium Composition on Their Growth

A total of 40 mg of the purchased culture of commercial microbial starter was aseptically transferred to previously prepared Vegitone infusion broth (Veg) or reference media (DRBC), both with volumes of 100 cm^3^. At 48 h after colonies of *G. candidum* or *P. camemberti* were grown on solid media, the material was collected with a swab and transferred to a test tube with saline to establish the appropriate culture density using a densitometer, i.e., 0.5 on the McFarland scale. Subsequently, 1 cm^3^ of the prepared suspension was transferred to 100 cm^3^ of liquid media Vegitone infusion broth (Veg) and Reference medium (DRBC). The cultivation lasted for 24 and 48 h on a reciprocating shaker at 140 rpm at temperatures of 28 and 37 °C. Serial dilution seeding was performed on Vegitone infusion with agar and DRBC media, and after 72 h at 28 °C, the grown colonies were counted and converted to log CFU/cm^3^.

Both purchased vegan bacterial cultures and strains from the culture collection were first multiplied and transferred to solid media. Twenty-four-hour colonies were transferred to sterile saline and adjusted to 0.5 McFarland using a densitometer. From the prepared suspensions, 10 µL was taken and transferred to 100 cm^3^ of liquid media MRS and MRS Vegitone (Veg MRS). The flasks were incubated in an incubator at 35 °C for 24 h. After incubation, serial dilution seeding was performed, utilizing solid media: MRS agar (MRS), M17 agar (M17), and MRS Vegitone Agar (Veg MRS) solidified with agar at a concentration of 20 g/dm^3^. After 24 h of incubation at 35 °C, the grown colonies were counted and converted to log CFU/cm^3^.

### 2.5. Plant-Based Camembert Cheese Analogues Preparation

Cheese alternatives with the compositions presented in Table 1 were prepared for the current study. The weighed ingredients were placed in the Thermomix TM6 bowl (Vorwerk, Wuppertal, Germany) and poured with the measured amount of hot water (in the ratio according to Table 1). In some variants, transglutaminase and calcium chloride were added at this stage. Some variants were prepared with the addition of transglutaminase enzyme Vzyme at a dose of 100 µL per one cheese analogue (Cashewbert, Berlin, Germany). Some variants were supplemented with calcium chloride at a dose of 0.6 g and 1.4 g CaCl_2_ per 100 g of plant mass. The mass was mixed to a smooth consistency, placed in a sterilized and dry bowl, and left to cool to a maximum temperature of 35 °C. Then, the selected strain of bacteria and mold fungi were added with the given proportions according to the manufacturer’s instruction, in the case of the Cashewbert cultures, one spoon (100 mg), or in the case of strains purchased from the Industrial Strains Collection, there was fresh-grown biomass prepared. LAB strains were cultured in Vegitone broth, centrifuged after 24 h, and added at a dose of the amount of biomass grown in 100 cm^3^ of medium to the portion of two cheese analogues.

Cylindrical boxes with baking paper were filled with the prepared mass to 2–3 cm height, closed, and left at room temperature for 4 h. Subsequently, boxes were placed in the refrigerator at 12 °C. During the first three days, the plant-based cheeses were gently turned over, and the excessive moisture was removed. Then, on the fourth day, they were salted on all sides. The cheeses were turned regularly to ensure their surfaces were evenly covered with white mold.

### 2.6. Physicochemical Analysis of Plant-Based Camembert Cheese Alternatives

The physicochemical analysis of cheese analogues assessed the fat, protein, and dry matter content using the FoodScan™ 2 apparatus (Foss Analytics, Warsaw, Poland). Moreover, the scope of the analysis was expanded to include the sodium chloride content and saturated fatty acids using the same device. To verify the obtained results, several cheese analogues were analyzed using traditional analytical methods, namely dry matter by drying method, fat content by hexane extraction in a Soxhlet apparatus and Bligh–Dyer method, and protein content by the Kjeldahl method [10]. The analyses were conducted for cheese analogues made from cashews, pistachios, peas, chickpea flour, and a mixture of cashews, soy flour, and spirulina.

Samples were freeze-dried and ground into powder before analysis. The total contents of C, N, and S were determined using an Elementar Vario MacroCube analyzer (Elementar, Langenselbold, Germany). The total contents of P, Na, K, Ca, Mg, Fe, Al, Mn, Cu, Zn, Ni, Sr, V, Sr, Ba, Pb, Co, and Cr were determined by the inductively coupled plasma atomic emission spectrometry—ICP-OES method using Avio 200 (Perkin Elmer, Waltham, MA, USA) after dissolving the samples in 65% HNO_3_.

### 2.7. Determination of Total Acidity Content of Plant-Based Camembert Cheese Alternatives

The total acidity content in the plant-based analogues was determined using titration with an automatic titrator TitraLab AT1000 Series (Hach, Wroclaw, Poland). In total, 1 g of the cheese analogue was weighed into the vessel in which the titration was performed and suspended in 50 cm^3^ of distilled water. Titration was performed using a 0.1 M potassium hydroxide (KOH) solution. The results were expressed as g of lactic acid/kg of cheese analogue.

### 2.8. Organoleptic Analysis of Plant-Based Camembert Cheese Alternatives

Organoleptic evaluation of the produced Camembert-type cheese analogues was conducted with the participation of 22 individuals. Using a 9-point structured hedonic scale (from 1 to 9, where 1 is the least desirable and 9 is the most desirable), three plant-based cheese alternatives were assessed for their appearance, color, sourness, saltiness, bitterness, creaminess, spreadability, aroma, and overall impression. Vegan cheese analogues chosen on the basis of chemo-physical analyses and preliminary selection (described in detail in the Section 3) were produced using the same starter culture of bacteria and mold, with cashews, pistachios, and a mixture of cashews, soy flour, and spirulina as the plant matrices. The preparation of Camembert-type cheese analogues involved a culture of LAB consisting of *L. lactis* KKP 3020 and *S. salivarius* KKP 3251, as well as the mold culture *G. candidum*.

### 2.9. Statistical Analysis

The conducted studies were performed in triplicate. The results were presented in bar charts with standard deviation. The obtained data underwent statistical analysis in the Statistica 13.3 program (TIBCO Software Inc., Palo Alto, CA, USA). Analysis of variance was conducted using the Brown–Forsythe test, and the normal distribution of data was checked using the Shapiro–Wilk test. Results were analyzed using one-way analysis of variance (ANOVA) and Tukey’s post hoc test for normally distributed and homogenous variance data or using the non-parametric Kruskal–Wallis test for remaining cases. The significance level was set at α = 0.05.

## 3. Results

### 3.1. Evaluation of Survival and Growth of Selected Strains of LAB and Molds in Vegan Media

**Figure 1:**
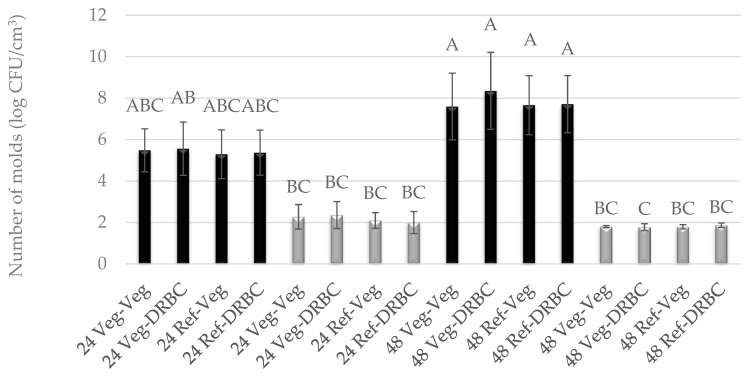
Number of *G. candidum* molds cultured for 24 h or 48 h in Vegitone vegan (Veg) and reference medium (DRBC) and inoculated onto vegan (Veg) and non-vegan agar medium (DRBC) (colonies were incubated at 28 °C—black bars or 37 °C—gray bars). Means with the same capital letter (A, B, etc.) did not differ significantly.

**Figure 2:**
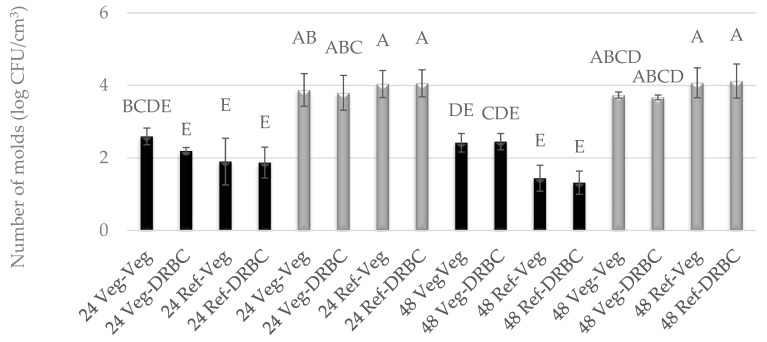
Number of *P. camemberti* molds cultured for 24 h or 48 h in Vegitone vegan (Veg) and reference medium (DRBC) and inoculated onto vegan agar medium Vegitone (Veg) and non-vegan medium (DRBC) (colonies were incubated at 28 °C—black bars or 37 °C—gray bars). Means with the same capital letter (A, B, etc.) did not differ significantly.

**Figure 3:**
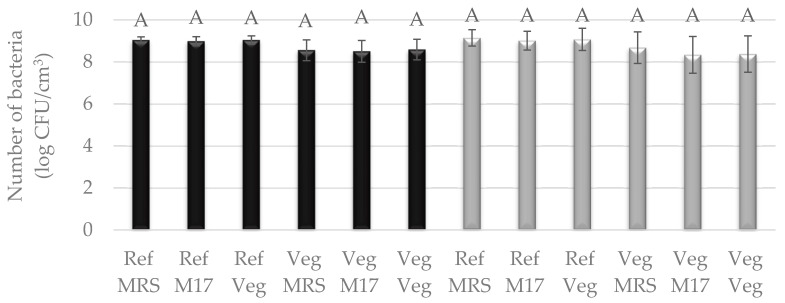
Number of LAB cultured in Vegitone vegan (Veg) and reference medium (MRS or M17) and inoculated onto vegan (Veg) and non-vegan media (MRS or M17) (mesophilic culture—black bars or thermophilic culture—gray bars). Means with the same capital letter (A) did not differ significantly.

**Figure 4:**
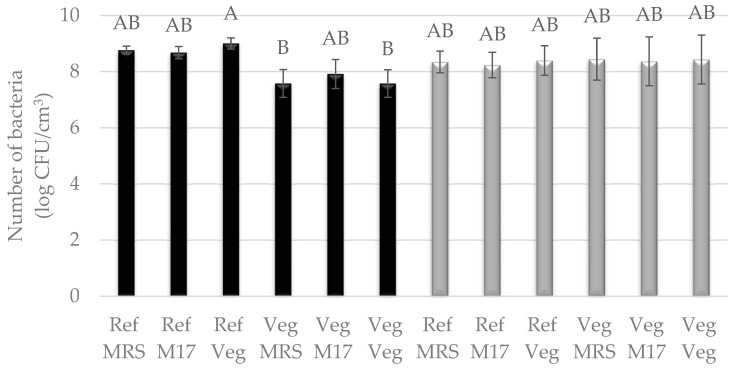
Number of LAB cultured in Vegitone vegan (Veg) and reference medium (MRS or M17) and inoculated onto vegan (Veg) and non-vegan media (MRS or M17) (*Lactococcus lactis* KKP 3020—black bars or *Streptococcus salivarius* KKP 3251—gray bars). Means with the same capital letter (A and B) did not differ significantly.

The production of vegan food products aims to eliminate raw materials and ingredients of animal origin not only in the final product intended for consumption but also throughout the entire product production process. In the case of food produced with microorganisms, it is necessary to replace traditional culture media, often containing animal protein hydrolysates or meat extracts, with plant equivalents. The first step of this study evaluated the growth and survival of selected strains of lactic fermentation bacteria and molds in culture media containing no animal-derived components (Figure 1, Figure 2, Figure 3 and Figure 4). Two selected commercial cultures of lactic fermentation bacteria and two pure LAB cultures, alongside two mold species, were multiplied in vegan media, including evaluation of their survival and growth intensity compared to commercially available reference media.

In the case of liquid media for molds, vegan Vegitone infusion broth and a mineral reference medium with 1% glucose were used. Solid media for molds included Vegitone infusion media with the addition of agar and DRBC. The growth of molds in Vegitone infusion broth and reference media was assessed after 24 and 48 h of cultivation at 28 and 37 °C. The cultures were inoculated using the serial dilution method on Vegitone infusion agar and DRBC media, and the obtained results are presented in Figure 1 and Figure 2. Both strains of mold were approved as potentially useful to produce plant-based cheese alternatives. Their growth in vegan and reference media was identical, with *G. candidum* growing better at 28 °C and *P. camemberti* at 37 °C.

For the mold of the *G. candidum* species, an increase in the number of cells was observed between 24 and 48 h of culture at 28 °C, regardless of the type of substrate used. The number of cells after 24 h reached an average level of 5.0 log CFU/cm^3^, and after 48 h, it ranged between 7.59 and 8.55 log CFU/cm^3^. At a temperature of 37 °C, weaker growth was observed at the level of 2.0 log CFU/cm^3^, and the number of cells did not increase between 24 and 48 h of culture (Figure 1). A similar observation can be described for the mold *P. camemberti* at a temperature of 28 °C. In turn, in the culture conducted at 37 °C, the number of cells was twice as high and reached the level of 4.0 log CFU/cm^3^ (Figure 2).

Bacteria were cultured in two types of media: liquid reference media, i.e., MRS and vegan modified MRS broth—Vegitone, and solid media: reference MRS agar and M17 agar and vegan MRS Vegitone agar. Bacterial growth in liquid media was assessed by inoculating 24 h cultures on solid media. The results for three cultures of LAB allowed the use of all of them in subsequent tasks for the production of cheese alternatives, because, as in the case of molds, no statistical differences were observed between their growth in vegan and reference media (Figure 3 and Figure 4). For the mesophilic culture, the number of bacteria was between 8.50 and 9.04 log CFU/cm^3^, and for the thermophilic culture, the number of bacteria was between 8.34 and 9.14 log CFU/cm^3^ (Figure 3). *S. salivarius* was grown at an average level of 8.0 log CFU/cm^3^. The exception was the *L. lactis* bacterial strain, which showed weaker growth by one logarithmic cycle in the vegan medium compared to the reference MRS, the highest number of cells was noticed for liquid reference media (8.68–9.00 log CFU/cm^3^), and the lowest for Vegitone (7.57 log CFU/cm^3^) (Figure 4).

### 3.2. Development of a Formulation for a Plant Analogue of Mold-Ripened Cheese

At the preliminary stage, the following plant-based raw materials for the preparation of plant-based Camembert cheese alternatives were tested: cashews, pistachios, soy flour, chickpea flour, pea protein, pumpkin protein, hemp protein, and spirulina. All were evaluated favorably for their use in the formulation of cheese alternatives. The result of this preliminary step was to select among the tested matrices which could be suitable for the preparation of plant-based Camembert cheese alternatives.

**Figure 5:**
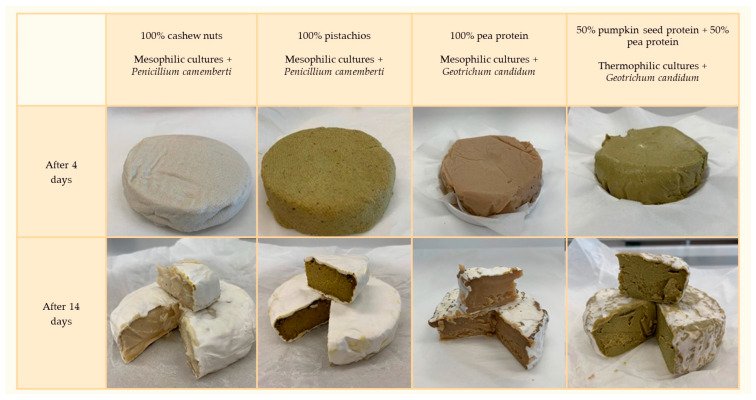

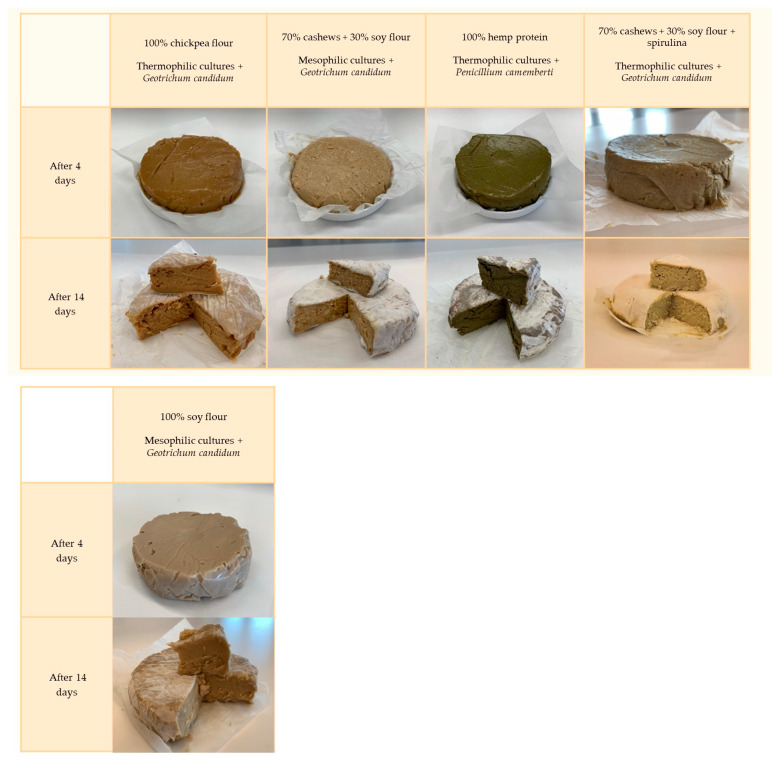
Ripening of Camembert cheese analogues depends on the plant matrix used and the starter cultures of LAB and mold.

Starter cultures were selected randomly. Several variants of cheese analogues were prepared using different combinations of two starter cultures of lactic fermentation bacteria and two species of filamentous fungi (a total of four different variations in the use of microorganisms, Table 2). The strains were added to the cheese mass. The ripening process was then carried out. Bacterial and mold growth were evaluated after 4, 7, 10, or 14 days of incubation at a temperature 12 °C (Figure 5). The optimal time required to cover cheese analogues with mold and to achieve the right balance between the bitter and sour taste of the final product was analyzed, which turned out to be a key factor influencing the final evaluation of the taste qualities of the analogue. All tested microorganisms present in commercial starter cultures showed good growth in plant matrices (Table 2), but individual combinations of microorganisms significantly affected the different flavors of the product. The lowest number of bacterial counts was noticed for the hemp analogue (7.07 log CFU/cm^3^), and the highest number was found in the soy flour product (10.04 log CFU/cm^3^). For other variants, the content of LAB varied from 8.96 log CFU/cm^3^ for the pumpkin and pea protein analogue to 9.19 log CFU/cm^3^ for the cashew products (Table 2).

After 14 days of incubation, the analogues were already fully covered with a white rind (Figure 5). The degree of rind coverage may be influenced by the matrix from which such an analogue was made. Comparing the results of cheese analogues made from pumpkin and pea seed protein, chickpea flour and cashews, soy flour, and spirulina, it can be seen that the same bacterial and mold cultures were used, while the results differed significantly. When cashews were incorporated into the mixture, the crust displayed the most appealing appearance. Looking also at the variants, where nuts accounted for 100% (cashews and pistachios) and for the one with a combination of cashews and soy flour, it can be seen that the cheese analogues were fully grown with a stately white rind, which may prove that the nut-based matrix may indeed be the most optimal choice. These results found reflection in mold counts which ranged from 4.11, 4.33, 4.87, and 4.96 CFU/cm^3^ for pea protein, chickpea flour, hemp protein, and pumpkin with pea protein analogues, respectively, to even 6.65 CFU/cm^3^ for pistachio products (Table 2).

**Figure 6:**
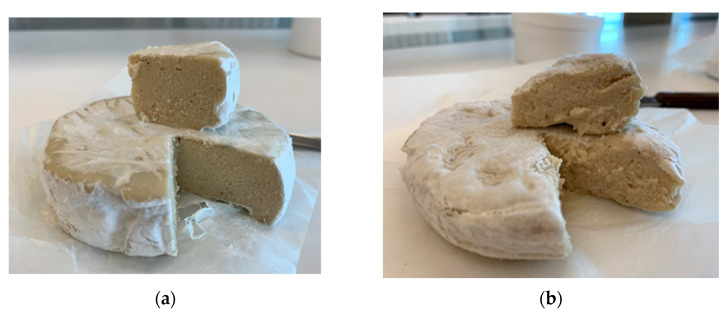
The impact of transglutaminase on cheese analogues prepared from cashews: (**a**) product with the addition of transglutaminase; (**b**) product without enzyme addition. Cashew mass was fermented with *L. lactis ssp. lactis*, *S. salivarius*, as well as *G. candidum* culture.

At the initial stage, the effect of the addition of transglutaminase on the physical properties of finished products made from cashew pulp was also investigated. The cashew pulp was chosen for transglutaminase addition because cashew-based cheese analogues were characterized by the least compact structure. Figure 6 shows the cross-sectional appearance of the products after 14 days of maturing. Although the final product prepared with the addition of the enzyme was characterized by better elasticity and uniformity, their effect on taste was negatively assessed, and this practice was no longer continued.

### 3.3. Physicochemical Analysis of Selected Plant-Based Camembert Cheese Alternatives

Several formulations for obtaining a Camembert cheese analogue (taking into account the starter culture of bacteria and molds, plant base, and ripening conditions) were selected for the next stages of the project: cashew-based alternatives, pistachios-, pea protein, and chickpea-based alternatives, as well as a mix of 70% cashew and 30% soy flour with spirulina. The selection of variants was made on the basis of a preliminary assessment of their appearance, aroma, and texture and color of the product. All investigated factors (analogue composition, LAB, and mold starter culture) did not influence the lactic acid content and pH of the final product, and the differences among analogue variants were statistically insignificant (Table 3). Lactic acid content varied from 10.74 g/kg for the pistachios analogue to 12.17 g/kg for the cashew, soy flour/spirulina variant, and pH ranged from 5.46 to 6.08. Analogues prepared with mesophilic commercial cultures contained, on average, 9.35 g/kg of lactic acid (pH 5.91), whereas analogues prepared with thermophilic LAB strains were characterized by 12.55 g/kg (pH 5.69).

**Figure 7:**
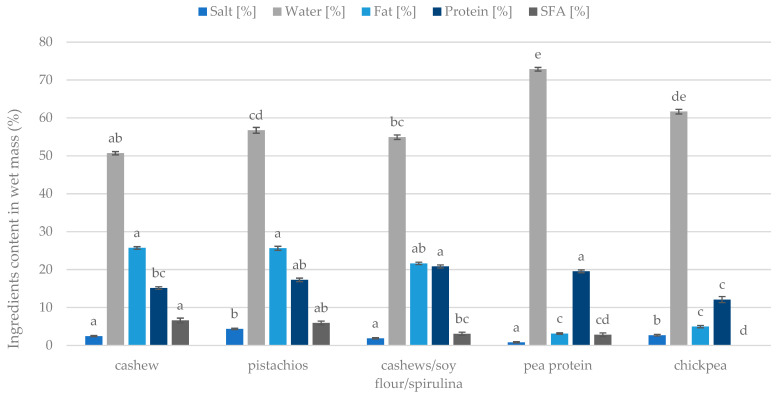
Composition of the plant analogues of Camembert cheese depending on the matrix used. Homogeneous groups are determined by the Kruskal–Wallis non-parametric test. Means with the same capital letter (a, b, ... etc.) did not differ significantly.

The contents of water, fat, proteins, salt, and saturated fatty acids are presented in Figure 7. The pea protein analogue was distinguished by having the highest water content, exceeding more than 70%, while the chickpea mass contained more than 60% water. At the same time, chickpea had the lowest protein content compared to the other masses (approx. 12%). As for saturated fatty acids, cashew and pistachio analogues present themselves as the samples with the highest amount of this component. Pistachios also stood out for having the highest salt content (Figure 7). Regarding fat content, cashews, pistachios, and the mixture of cashews, soy, and spirulina showed the highest fat content compared to the mass from peas or chickpeas. Plant alternatives varied in main compounds concentration; it could be noticed that chickpea, cashew, and pistachios analogues differed the most and were chosen for the next experiments.

Element content of non-supplemented cheeses prepared from five different matrices—chickpeas, cashews, pistachios, peas, and a mixture of cashews and soy flour with spirulina—was evaluated (Table 4). All analogues, except pistachio-based analogues, were prepared with a mold culture of the genus *Geotrichum*. Pistachio-based analogues were made with the participation of a mold of the *Penicillium* genus. For the preparation of analogues of Camembert-type cheeses, lactic fermentation bacterial cultures consisting of *Lactococcus lactis* KKP 3020 and *Streptococcus salivarius* KKP 3251 were used, except for analogues based on a mixture of cashew, soy flour, and spirulina, which were prepared with the participation of a thermophilic culture of *St. thermophuilus*, *Lactobacillus helveticus*. The maturation time of the products was 14 days.

The results in Table 4 present the content of elements in various types of cheese alternatives. The greatest attention has been focused on calcium because it is important in the human diet devoid of dairy products, which are the main source of this element. The cheese analogues made from chickpeas and pistachios contained the highest concentration of calcium, i.e., 1.36 and 1.25 g/kg, while those made from cashews contained the least (0.39 g/kg).

Vanadium, cobalt, and arsenic were not detected in any of the samples tested. The matrices of plant analogues of Camembert cheese differed in other elements’ content. The highest carbon content was found in the pistachio variant (64.81 g/kg), with the lowest for the chickpeas variant (46.96 g/kg), while the nitrogen content was highest in the pea variant, and the lowest was only 34.09 g/kg for cashews. The analogues differed in sodium content (15.79 g/kg for the cashews analogue and just 0.06 g/kg for the chickpeas variant). Large disparities in elemental content between the variants also concerned iron (6.4 mg/kg for pistachios and 196.0 mg/kg for chickpeas) and aluminum (which was absent in the cashews and pistachios analogues).

It could be seen that chickpea-based and pea protein-based analogues contained low amounts of lipids and, in relation to the chickpea analogue, also low protein content when compared to cashew, pistachios, and cashew/soy flour cheese analogues. High aluminum content of chickpea variants also did not encourage us to select this variant for further steps. On the basis of chemical composition, only three mold-ripened cheeses were further investigated.

### 3.4. Fortification of Camembert Cheese Plant Analogues with Calcium Correlated to Composition of Basic Elements

The fortification of plant-based cheese analogues with calcium, ensuring the appropriate calcium concentration, was also assessed. Using data on the calcium content of the cheese analogues (Table 4), two doses of calcium in the form of calcium chloride were selected and applied to three plant variants of Camembert cheese alternatives (cashew-based, pistachio-based and chickpea-based). A dose of 0.6 g and 1.4 g of CaCl_2_ was added for each 100 g of plant matter, which corresponded to a calcium dose of 0.22 and 0.51 g per 100 g of product, respectively.

Cheese analogues prepared with chickpea flour contained 1.4 g Ca/kg for the unsupplemented product and up to 2.8 g Ca/kg when supplemented with a higher dose of calcium chloride (Table 5). In pistachio and cashew products, this value was 1.3 g Ca/kg and 0.4 g Ca/kg in control samples and up to 1.8 g Ca/kg and 1.3 g Ca/kg in supplemented samples, respectively. In pistachio nuts themselves, as the raw material used to prepare cheese analogs, there was a high salt content (understood as a high concentration of sodium and chlorine ions). Hence, the target assumption was that when using a form of calcium supplementation as calcium chloride, only the lower assumed dose of calcium would be used.

Calcium supplementation raised the sodium content determined in the final product, probably because of contamination of calcium chloride with sodium salts. In addition, heavy metals were found to be absent in the cheese analogues (Table 6). Other differences in macro- and micro-element content were similar or close to those obtained in the previous stage of the study (Table 4).

### 3.5. Organoleptic Analysis of Plant-Based Camembert Cheese Alternatives

**Figure 8:**
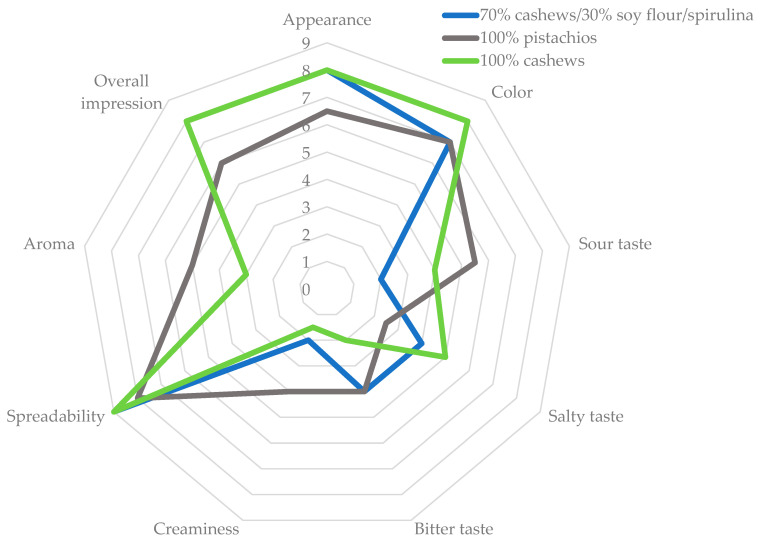
Organoleptic evaluation of plant-based analogues of Camembert cheese.

The graph shows the results of the organoleptic evaluation of plant-based cheese analogues, based on a hedonic scale from 0 to 9, where respondents rated different aspects of the variants (Figure 8, Table 7). The observations show that in terms of spreadability and color, all variants received similar ratings. On the other hand, taste, in particular sweet, salty, and sour, evoked different impressions depending on the specific variant. The aroma of the analyzed analogues was rated relatively low, oscillating between 2 and 4, as was creaminess, which scored even lower, ranging between 1 and 3. As for appearance, all variants were rated between 6 and 8 points. In the overall impression category, the pistachio-based variant received a score of 6, while the other two variants were rated at 8, and the difference was statistically significant. On the basis of organoleptic analysis, it can be predicted that prepared Camembert cheese alternatives might be acceptable for consumers.

## 4. Discussion

Maturation of all mold cheeses is based on surface yeasts and molds, but many varieties are produced using different cheesemaking practices and formulations. The fungi most commonly used include *P. candidum* (*P. camemberti*), *G. candidum*, and *Kluyveromyces marxianus*. These yeasts and molds, in addition to their obvious function of ripening cheese, also give it its characteristic white-grey appearance [11]. Usually, the maturation processes initiate at higher temperatures to encourage white noble mold growth, and then they are cooled to around 10 °C to slow ripening. These steps include the hydrolysis of proteins and fats, which contribute to the smooth and creamy texture inside the product, the production of aroma and flavors, and primarily the white rind covering the cheese matrix. Furthermore, the benefits of the presence of LAB and the production of organic acids (e.g., lactic and acetic acids) lead to a lower pH value and enhanced fungal growth [12].

The species of *G. candidum* and *P. camemberti* have the greatest impact on the flavor and texture of moldy cheeses. Studies have shown that *G. candidum* reduces bitterness and affects rind thickness, texture, and firmness [13]. An aspect that also has great importance on the sensory characteristics of the final product is the type of lactic bacteria. Cheese-making lactic bacteria are divided into two basic groups: mesophilic and thermophilic. The former grow well at 30–32 °C, while thermophilic ones grow at around 52 °C [14]. In dairy products, LAB are responsible for converting lactose into lactate during milk fermentation, which is then consumed by yeast and also lowers the pH. The population of starter microorganisms dominates during the initial period of production and gradually reduces under the influence of changing environmental factors during the ripening process [15]. Similar to the fungi present in the rind cheeses, LAB fermentation also promotes several secondary changes, such as changes in texture and nutritional value, as well as flavor production. All these changes are strongly dependent on the metabolic activity of the fermenting bacteria and the chemical composition of the food matrix. Research showed that LAB can exhibit product and health benefits not only in dairy products. An example is cashew juice. Although not a dairy product, lactic fermentation has developed a variable profile combining cashews and volatiles from lactic acid fermentation with mild formation or decomposition of aromatic compounds [16].

The possibility of culturing microorganisms, especially LAB, in plant-derived nutrient media will be of great interest to specific consumer communities. It may also pave the way for the production of “animal-free” probiotics and other products suitable for the vegan diet. Several studies have been concerned with elaborating plant-based media for LAB [17,18] and other microorganism cultures [19,20]. Parecha et al. [17] applied the Design of Experiments to optimize a growth medium based only on vegan components in the cultivation of the probiotic strain *Lactobacillus paracasei* IMC502^®^. The authors compared different carbon and nitrogen sources in the self-made media, considering glucose, maltose, sucrose, and galactose as carbon sources. Wheat, soya, potato, rice, and guar peptones were used instead of beef extract and proteose peptone in the MRS medium. Interestingly, the application of all the tested nitrogen sources remarkably increased the biomass production of *L. paracasei*, but the authors paid attention to guar peptone, which is not a part of the human diet and, hence, will not affect the increase in prices of other plant proteins. Finally, biomass production increased by about 65% in comparison to the plain MRS medium [17]. Considering vegan or vegetarian food, Ayu et al. [18] take into account also halal food, where products manufactured with microorganisms must be devoid of all ingredients that come from prohibited sources under Islamic law. This resulted in the desire to look for an alternative medium for cultivating LAB. The authors developed a plant-based medium consisting of sweet potato extract with 10 g/L soybean powder. Similar to the current study, the vegan media were suitable for cultivating LAB, where *Lactiplantibacillus plantarum* TISTR 2075 exhibited no significant difference in the cell number in comparisons between plant-based and MRS media (8.94 vs. 9.05 log CFU/cm^3^) [18].

In addition, “plant-based beverages” provide a very good basis for the growth of microorganisms that exhibit probiotic activity due to the presence of indigestible components with prebiotic properties, such as fiber, inulin, and β-glucans. In this way, starch and dietary fiber improve physical stability and promote the survival of starter cultures in the digestive tract. Pistachio-based beverages are suitable media for the growth and survival of LAB. The authors demonstrated the growth and viability of bacterial cells over a 30-day storage period, resulting in a slightly decreased bacterial count compared to the 1-day for MA400 and MY800 starter cultures [21].

Interestingly, attempts to obtain a Camembert analogue were made by Łopusiewicz et al. [22]. The study focused on utilizing flaxseed oil cake as a plant-based medium and fermenting it with LAB and fungi, specifically *P. camemberti* and *G. candidum*. Both LAB and fungi grew in this matrix, and the highest number of cells was observed at 14 days of storage, with approximately 8 × 10^7^ and 1 × 10^6^ CFU/g, respectively. Then, a one-log decrease in the microorganism count was observed for up to 28 days of storage. What is important is that the flaxseed oil cake was a source of polyphenolic compounds with high antioxidant activity, preserved after fermentation and storage, indicating the receipt of a valuable food product [22]. The cited research, as well as the results obtained in this publication, confirm the possibility of using seeds and nuts to obtain Camembert cheese analogues while maintaining their antioxidant potential, high content of bioactive compounds, and the possibility of growing both LAB and fungi. Both studies showed that lactic acid, an organic carboxylic acid and a characteristic secondary metabolite of the starter cultures used, was produced. In the current study, the production of lactic acid was not dependent on the medium or strains used, but it was significantly higher than that observed for the fermented flaxseed oil cake. The highest observed concentration of lactic acid was 1.26 ± 0.18 g/100 g compared to 0.80 ± 0.01 g/100 g [22]. Furthermore, compared to milk-based yoghurt, cashew had similar physicochemical properties, including viscosity and pH. However, it had a relatively lower titratable acidity (TA). Cow’s milk contains about 4.6% lactose, but lactose does not occur naturally in cashew nuts. LAB are not expected to produce organic acids as a result of lactose fermentation in cashews, which may explain the lower TA in cashews. However, the lack of lactose did not prevent the accumulation of LAB to probiotic levels [23]. It should be noted that our own results are in line with the cited authors, and the growth of lab starter cultures was significant.

In the current study, Camembert cheese analogues were fortified with calcium ions in the form of calcium chloride. Inadequate calcium intake in the diet can contribute to the development of diseases such as osteoporosis and hypertension, but it can also play a role in the prevention of breast, prostate, and colon cancer [24]. For comparison, Camembert cheeses contain an average of 0.4 g of calcium, natural yoghurt 0.1 g, and Swiss almond cheese spread 0.6 g (nutritional values of the compared products were obtained from the U.S. Department of Agriculture and refer to a 100 g serving size). The results presented here indicate the presence of approximately 3 g/100 g of calcium in chickpea cheese analogues and about 1.5 g/100 g for pistachio and cashew analogues. Therefore, it can be concluded that vegan Camembert cheese analogues might be a good alternative to dairy products and can meet the recommended daily calcium intake for adults.

The created plant analogues were satisfactory in appearance and taste but differed significantly in these characteristics from dairy products. Detailed analyses of one study in which flaxseed oil cakes were used to create a plant-based version of Camembert cheese confirmed the sentiments of researchers and participants in organoleptic analysis of plant-based alternatives—their taste and flavor profile, as well as texture, turned out to be similar to real ones. Water loss and plant cell wall degradation during the ripening process, as well as the protein content and, thus, protein gelation properties in the plant matrix played crucial roles in texture formation [22]. Furthermore, the desired structure can be obtained using transglutaminase, which was also evaluated in this study. Transglutaminase catalyzes the crosslinking of glutamine and lysine residues, resulting in the formation of high molecular weight proteins with new beneficial properties. These properties include gelation, increased viscosity, and water-holding capacity. This enzyme was also applied in dense plant-based matrices used for the production of meat analogues [25] and burger patties [26]. The authors of both studies found that the final product properties were dependent on the transglutaminase concentration, concluding that it may be useful for the formation of the fibrous structure of plant proteins and support the production of clean-label food [25,26]. On the other hand, properties such as gumminess, chewiness, and hardness increased for the burger patties in comparison with meat analogues without enzyme treatment [26].

Edible nuts like pistachios or cashews are rich sources of lipids and proteins and contain on average 45.09% and 43.71% lipids and 19.80% and 18.81% proteins, respectively. Regardless of seed type, lipids are mainly composed of unsaturated fatty acids and do not contain cholesterol [27]. There are not many data on mineral contents of plant sources used in this study to produce cheese analogues, but it is predicted that the amount of such research will be growing as the consumption of plant-based foods that are marketed as alternatives for milk is increasing among consumers and its content needs to be compared to milk [28]. Harmankaya et al. [29] determined by ICP-AES mineral contents in pistachio kernels. The maximum values of K, P, Ca, Mg, and S elements achieved 8.064 mg/kg, 5.228 mg/kg, 3.226 mg/kg, 2.402 mg/kg, and 1.825 mg/kg, respectively, in kernels. In addition, the mean values of Fe, Zn, Cu, Mn, B, Mo, Cr, and Ni elements were determined. These studies indicated that those nuts contain high amounts of potassium, which is in agreement with our own results.

Redan et al. [28] analyzed eight plant-based milk alternatives produced from almond, cashew, coconut, hemp, oat, pea, rice, and soy. The results showed that pea milk alternatives contained the highest mean amounts of phosphorus, selenium, and zinc, while soy-based products were highest in magnesium. In comparison to our own results, cadmium and lead were found at low or non-quantifiable amounts [28]. In Camembert cheese analogues, unlike in cited research, there was no selenium identified in samples. Very high concentrations of sodium in nut-based cheese analogues were confirmed by Tošic et al. [30], who analyzed 19 elements in nuts (e.g., walnuts, hazelnuts, cashews, peanuts, and pistachios) and evaluated the descending order of macro-elements: Na > Mg > Ca > K. What should be noted is much higher contents of potassium in cashew-based and pistachios-based alternatives of Camembert analogues.

It was interesting that no aluminum was identified in cashew and pistachio alternatives, but it was analyzed in high concentrations in chickpea products. Akbaba et al. [31] found out that the pistachio samples grown under a conventional farming regime could contain harmful metals like Al. High contents of some metals are related to the origin of plant substrate [31]. Moreover, it was proven that acidic soils impact higher Al absorption by plant roots [32].

The advantage of the prepared products is undoubtedly that they are intended for consumers who follow a vegan and vegetarian diet, for whom there is still little variety on the market. The proposed analogs, although mimicking mold-ripened cheese, could also be a separate range of food products. Moreover, in the long run, they could be a source of probiotic microflora after appropriate selection of starter culture of lactic acid bacteria. These are products with high nutritional value, rich in protein and unsaturated fatty acids, and could stand as a source of magnesium or zinc (when describing cashew-based products). 

## 5. Conclusions

In conclusion, after testing various raw materials, it was found that pistachios and cashews are the best options for creating plant-based alternatives to Camembert cheese. Other materials like chickpea flour and pea protein, while high in protein, did not meet expectations in terms of taste. Pistachio and cashew-based analogues were characterized with the best taste and appearance and appeared to be good matrices for mold and lactic acid bacteria starter cultures. Those analogues might stand for fat and protein sources in a balanced diet as well as some macro- and microelements.

It is important to note that the aim of this research was not just to replicate Camembert cheese but to use its methods to create a new product with a unique flavor. It is also suggested to consider a new name for these alternatives, moving away from the term “vegan cheese”, e.g., Nutmbert or Plantmbert. This could attract a wider customer base, as those on vegan diets often seek novelty, while non-vegan consumers may avoid products labeled “vegan”. The results of this research are an important step in developing innovative food products for consumers who want to avoid animal products. These plant-based cheese alternatives offer new options for vegetarians and vegans, improving the range and quality of plant-based diet choices. This study also includes valuable analyses comparing the micro-nutrient compositions of traditional dairy products with their plant-based counterparts.

## Figures and Tables

**Table 1:** Cheese alternative compositions used in this study.

No.	Plant Base	Plant Base: Water Ratio
1	100% Cashew nuts	1:1
2	100% Pistachio nuts	3:2
3	100% Pea protein	1:3
4	100% Hemp protein	1:1
5	50% Pumpkin protein + 50% pea protein	2:3
6	100% Soy flour	1:1
7	100% Chickpea flour	1:1
8	70% Cashew nuts + 30% soy flour	1:1
9	70% Cashew nuts + 30% soy flour + 0.5% spirulina powder	1:1

**Table 2:** Number of LAB and molds after 14 days of ripening for the selected Camembert cheese analogues.

Plant Base	Bacterial Starter Culture ^1^	Mold Starter Culture ^2^	LAB (log CFU/cm^3^)	Molds (log CFU/cm^3^)
100% Cashews	M	P	9.19 ± 0.20	5.27 ± 0.14
100% Pistachios	M	P	9.18 ± 0.10	6.65 ± 0.13
100% Pea protein	M	G	8.77 ± 0.15	4.11 ± 0.23
50% Pumpkin protein + 50% pea protein	T	G	8.96 ± 0.12	4.96 ± 0.08
100% Soy flour	T	G	10.04 ± 0.40	5.40 ± 0.18
70% Cashew nuts + 30% soy flour	M	G	9.08 ± 0.21	5.33 ± 0.32
100% Hemp protein	T	P	7.07 ± 0.13	4.87 ± 0.10
100% Chickpea flour	T	G	7.84 ± 0.20	4.33 ± 0.15

^1^ M—mesophilic or T—thermophilic commercial starter culture; ^2^ P—*P. camemberti* or G—*G. candidum* commercial starter culture.

**Table 3:** Titratable acidity and pH of plant analogues of Camembert cheese after 14 days of ripening expressed in lactic acid content.

Plant Matrix	Lactic Acid Content (g/kg)	pH
Cashews	10.04 ± 0.34	5.51 ± 0.11
Pistachios	10.74 ± 1.09	5.46 ± 0.08
Cashews/soy flour/spirulina	12.17 ± 1.53	5.74 ± 0.24
Pea protein	11.78 ± 2.64	6.08 ± 0.46
Chickpea	10.88 ± 0.50	5.59 ± 0.12
**LAB Starter Culture**	**Lactic Acid Content (g/kg)**	**pH**
Mesophilic commercial culture	9.35 ± 1.14	5.91 ± 0.17
Thermophilic commercial culture	12.55 ± 1.83	5.69 ± 0.32
culture composed of *L. lactis* and *S. salivarius*	11.82 ± 0.68	5.51 ± 0.12
**Mold Starter Culture**	**Lactic Acid Content (g/kg)**	**pH**
*G. candidum*	11.48 ± 0.74	5.55 ± 0.12
*P. camemberti*	10.56 ± 0.94	5.80 ± 0.16

Parameters were compared based on three parameters: plant matrix, introduced lactic acid starter culture (mesophilic commercial culture, thermophilic commercial culture, and culture composed of *L. lactis* and *S. salivarius*), and mold species (*G. candidum* and *P. camemberti*). Means ± standard error are included. Means are expressed as weighted means.

**Table 4:** The content of calcium and other elements in plant analogues of Camembert cheese depending on the plant matrix used.

Elements	Unit	Chickpeas	Cashews	Pistachios	Peas	Cashews/Soy Flour/Spirulina
C	g/kg	46.96	59.73	64.81	51.78	57.48
N	g/kg	39.63	34.09	41.49	130.70	50.29
S	g/kg	2.36	1.57	1.36	2.85	2.20
P	g/kg	3.17	3.54	3.58	8.05	5.18
Na	g/kg	0.06	15.79	2.69	9.73	4.73
K	g/kg	10.48	5.83	10.06	2.86	10.51
Ca	g/kg	1.36	0.39	1.25	0.81	0.96
Mg	g/kg	1.49	2.12	1.32	0.47	3.03
Fe	mg/kg	196.0	21.9	6.4	184.8	67.8
Al	mg/kg	223.8	0.0	0.0	7.6	14.0
Mn	mg/kg	30.70	11.25	11.90	10.33	24.00
Cu	mg/kg	17.34	22.93	7.21	9.38	24.82
Zn	mg/kg	26.81	38.46	17.22	64.53	51.90
Ni	mg/kg	0.44	2.36	0.00	0.00	4.72
Pb	mg/kg	0.88	0.04	0.00	0.08	0.29
Sr	mg/kg	10.99	1.51	21.42	12.98	3.67
Ba	mg/kg	2.11	0.58	0.09	2.06	1.72

**Table 5:** Content of calcium and other macronutrients in plant analogues of Camembert cheese supplemented and not supplemented with calcium chloride.

Maturation Time (Days)	CaCl_2_ (g/100 g)	Plant Matrix	C	N	S	P	Na	K	Ca	Mg
			(g/kg)							
14	0	chickpeas	46.96	39.63	2.36	3.17	8.06	10.48	1.36	1.49
7	0.6		45.63	38.74	1.80	2.80	8.62	9.56	1.78	1.40
7	1.4		45.59	38.25	2.01	2.98	8.88	9.93	2.91	1.45
14	0.6		46.02	38.56	1.58	2.66	8.30	9.51	1.89	1.36
14	1.4		44.84	37.61	1.73	2.93	14.10	9.51	2.84	1.45
14	0	cashews	59.73	34.09	1.57	3.54	15.79	5.83	0.39	2.12
7	0.6		59.90	36.00	1.58	4.19	7.14	5.84	1.50	2.53
7	1.4		59.57	36.80	1.47	3.92	6.90	5.48	1.07	2.40
14	0.6		59.81	35.89	1.31	3.96	8.35	5.39	0.91	2.45
14	1.4		58.26	35.35	1.33	3.97	15.96	5.56	1.29	2.44
14	0	pistachios	64.81	41.49	1.36	3.58	2.69	10.06	1.25	1.32
7	0.6		62.25	36.96	1.30	2.86	15.19	6.22	1.81	1.04
14	0.6		62.56	36.59	1.18	2.51	18.66	6.37	1.47	0.94

**Table 6:** The micronutrient content of plant analogues of Camembert cheese supplemented and not supplemented with calcium chloride.

Maturation Time (Days)	CaCl_2_ (g/100 g)	Plant Matrix	Fe	Al	Mn	Cu	Zn	Ni	Pb	Sr	Ba
			(mg/kg)								
14	0	chickpeas	196.0	223.8	30.70	17.34	26.81	0.44	0.88	10.99	2.11
7	0.6		157.4	182.6	28.08	10.16	24.31	0.00	0.41	10.27	2.40
7	1.4		171.0	186.9	29.40	11.21	26.56	0.35	0.10	10.89	3.38
14	0.6		176.7	207.5	25.08	9.52	21.69	0.03	0.25	10.52	2.50
14	1.4		162.7	186.6	28.49	10.14	24.69	0.00	0.37	11.08	3.49
14	0	cashews	21.9	0.0	11.25	22.93	38.46	2.36	0.04	1.51	0.58
7	0.6		28.4	0.0	16.58	26.34	49.22	3.99	0.06	1.89	1.65
7	1.4		27.9	0.0	15.80	25.33	47.21	3.55	0.21	1.68	1.16
14	0.6		33.8	0.0	16.74	25.38	50.77	3.68	0.07	1.64	1.04
14	1.4		29.3	0.0	15.83	25.46	49.22	3.51	0.22	1.83	1.44
14	0	pistachios	6.4	0.0	11.90	7.21	17.22	0.00	0.00	21.42	0.09
7	0.6		0.0	0.0	7.30	4.95	11.05	0.00	0.46	21.95	1.19
14	0.6		2.7	0.0	6.10	5.42	9.95	0.00	0.00	20.92	0.66

**Table 7:** Organoleptic evaluation of plant analogues of Camembert cheese (median response).

Plant Matrix	70% Cashews/30% Soy Flour/Spirulina	Cashews	Pistachios
Appearance *	8	6.5	8
Color *	7	7	8
Sour taste	2 ^a^	5.5 ^b^	4 ^ab^
Salty taste	4 ^ab^	2.5 ^a^	5 ^b^
Bitter taste *	4	4	2
Creaminess	2 ^ab^	4 ^b^	1.5 ^a^
Spreadability	9 ^ab^	8 ^a^	9 ^b^
Aroma	3 ^a^	5 ^b^	3 ^ab^
Overall impression	8 ^ab^	6 ^a^	8 ^b^

The lack of a statistically significant difference is marked with *; homogeneous groups determined based on the Kruskal–Wallis test. Means with the same capital letter (a and b) did not differ significantly.

## Data Availability

The original contributions presented in the study are included in the article, further inquiries can be directed to the corresponding author.
